# microRNA expression profile of peripheral blood mononuclear cells of Klinefelter syndrome

**DOI:** 10.3892/etm.2012.682

**Published:** 2012-08-24

**Authors:** WEIGUO SUI, MINGLIN OU, JIEJING CHEN, HUAN LI, HUA LIN, YUE ZHANG, WUXIAN LI, WEN XUE, DONGE TANG, WEIWEI GONG, RUOHAN ZHANG, FENGYAN LI, YONG DAI

**Affiliations:** 1 Guangxi Key Laboratory of Metabolic Disease Research, Central Laboratory of Guilin 181st Hospital, Guilin 541002;; 2 Key Laboratory of Laboratory Medical Diagnostics, Ministry of Education, Chongqing Medical University, Chongqing 400016;; 3 Clinical Medical Research Center of The Second Clinical Medical College, Jinan University, Shenzhen People’s Hospital, Shenzhen 518020, P.R. China

**Keywords:** deep sequencing, Klinefelter syndrome, microRNA

## Abstract

microRNAs are a type of small non-coding RNAs which play important roles in post-transcriptional gene regulation, and the characterization of microRNA expression profiling in peripheral blood mononuclear cells (PBMCs) from patients with Klinefelter syndrome requires further investigation. In this study, PBMCs were obtained from patients with Klinefelter syndrome and normal controls. After preparation of small RNA libraries, the two groups of samples were sequenced simultaneously using next generation high-throughput sequencing technology, and novel and known microRNAs were analyzed. A total of 9,772,392 and 9,717,633 small RNA reads were obtained; 8,014,466 (82.01%) and 8,104,423 (83.40%) genome-matched reads, 64 and 49 novel microRNAs were identified in the library of Klinefelter syndrome and the library of healthy controls, respectively. There were 71 known microRNAs with differential expression levels between the two libraries. Clustering of over-represented gene ontology (GO) classes in predicted targets of novel microRNAs in the Klinefelter syndrome library showed that the most significant GO terms were genes involved in the endomembrane system, nucleotide binding and kinase activity. Our data revealed that there are a large number of microRNAs deregulated in PBMCs taken from patients with Klinefelter syndrome, of which certain novel and known microRNAs may be involved in the pathological process of Klinefelter syndrome. Further studies are necessary to determine the roles of microRNAs in the pathological process of Klinefelter syndrome in the future.

## Introduction

Klinefelter syndrome is the most common sex-chromosome disorder in males with a prevalence of approximately 1 in 600, and is defined as a male having a karyotype containing an extra X-chromosome (47, XXY) and variants including mosaicims (1). Since the first description of Klinefelter syndrome in 1942, Klinefelter syndrome (47, XXY) has been known as a relatively common cause of infertility, hypergonadotropic hypogonadism, gynaecomastia and learning disability in males (2). Certain studies have predicted that the aberrant expression of X-chromosome-linked genes plays a key role in those clinical features of Klinefelter syndrome, but the mechanisms remain poorly understood, and its treatment is difficult and rarely successful (3). microRNAs are an abundant class of highly conserved, small non-coding RNAs that present a new theme of post-transcriptional gene regulation (4). Advances in understanding of the molecular events underlying types of human diseases, including virology, embryogenesis, differentiation, inflammation and cancers, have spurred numerous investigations to examine the comparative expression of microRNAs (5–8). However, few studies have revealed or focused on the character of microRNAs in Klinefelter syndrome until now.

The early attempts at systematically profiling microRNA expression were performed using detection experiments, including northern blotting, real-time PCR and complementary DNA (cDNA) microarrays (9). However, these methods lack the ability to identify novel microRNAs and accurately determine expression at a range of concentrations (10). Recently, studies have documented that numerous new microRNA annotations originate from deep-sequencing experiments (11). Before the effect of microRNAs on gene regulation can be globally studied in Klinefelter syndrome, a robust method for profiling the expression level of each microRNA in this disorder is required (12). However, information in this field was unavailable until now. Therefore, in order to obtain more basic information with regard to microRNAs in Klinefelter syndrome, our objective in this study was not to analyze the effect of microRNAs on gene regulation, but aim to profile and identify changes in microRNA expression in Klinefelter syndrome by high-throughput sequencing technology.

## Materials and methods

### Small RNA library preparation and sequencing

All patients and healthy controls were recruited at the Guilin 181st Hospital (Guilin, China), and written informed consents were obtained from all subjects. Seven blood samples were obtained from the patients who were diagnosed with Klinefelter syndrome with a karyotype containing an extra X-chromosome (47, XXY), and seven from healthy volunteers. This study was performed in accordance with the provisions of the Declaration of Helsinki 1995 (as revised in Edinburgh 2000).

Approximately 5 ml heparinized venous blood sample was obtained from each subject, peripheral blood mononuclear cells (PBMCs) in each blood sample were separated using Ficoll-Paque (Sigma, St. Louis, MO, USA) and RNA was extracted using TRIzol reagent (Invitrogen, Carlsbad, CA, USA) according to the manufacturer’s instructions. Subsequently, the extracted RNA was stored at −80°C until the preparation of small RNA libraries.

To produce the Klinefelter syndrome library and healthy control library, aliquots of total RNA from each subject were subjected to microRNA library construction and sequencing. The procedure was described previously (13,14). Briefly, RNA fragments 18–30 bases long were isolated from total RNA by 15% PAGE gel. A 5′ adaptor was ligated to purified small RNAs followed by purification of ligation products by 15% PAGE gel. The 3′ RNA adapter was subsequently ligated to precipitated RNA using T4 RNA ligase, followed by purification of ligation products by 10% PAGE gel. The ligation products were reverse transcribed and subjected to RT-PCR (Superscript II reverse transcriptase, 14 cycles of amplification) amplification. Amplification products excised from 6% PAGE gel were used for clustering and sequencing by Illumina Genome Analyzer (BGI, Shenzhen, China).

### Initial processing of the reads

The sequence tags from high-throughput sequencing underwent data cleaning, which included filtering certain low quality reads according to base quality value, trimming the adaptor sequence at the 3′ primer terminus and removing 5′ adaptor contaminants formed by ligation. Second, each high-quality clean reads >18 and <30 nt was aligned to the human reference genome (hg19, National Center of Biotechnology Information build 37.1) using the SOAP program (15). Third, sequences perfectly matched over their entire length were considered for further analyses. Certain annotated RNAs were discarded, including rRNA, scRNA, snoRNA, snRNA, piRNA, and tRNA deposited at NCBI GenBank database and Rfam 9.1 database; repeated overlapping sequences and the sequences overlapping with predicted exons and introns were also filtered. The remaining unique small RNA sequences were aligned with miRBase 14.0 database, novel microRNA candidates in the two groups of samples were predicted by Mireap and the procedure was conducted as previously described (13,16).

### Differentially expressed patterns of known microRNAs

Relative expression analysis which was normalized by accounting for the number of microRNAs and the total number of small RNA reads was sought to define the expression preferences of individual microRNAs between these two libraries. The procedures were as previously described (14), and briefly as follows: i) read counts of each identified microRNA in each library were normalized to the total number of small RNA reads, the ratio was then multiplied by a constant of 1×10^6^; ii) fold-change and P-value were calculated from the normalized expression: log_2_ fold-change and Audic-Claverie method were used to define differential expression of microRNAs between the two small RNA libraries, with a minimum fold-change (log_2_ ratio) of 1 and a false discovery rate cut-off of 0.01. The P-values were calculated according to the following equation:

$$p(x|y)={\left(\frac{N_{2}}{N_{1}}\right)}^{y}\frac{(x+y)!}{x!y!{\left(1+\frac{N_{2}}{N_{1}}\right)}^{\left(x+y+1\right)}}$$

where x indicates the number of reads across a microRNA in Klinefelter syndrome and y indicates the number of reads across the corresponding microRNA in the healthy control. In this study, the P-value indicates the probability of obtaining y counts in Klinefelter syndrome given x counts in healthy controls. In normalized sequence counts, a 1-fold change (log_2_ ratio) and P≤0.001 were considered to indicate a statistically significant result.

### Functional annotation of novel microRNA targets

The target genes of novel microRNAs in the two libraries were predicted, and the prediction rules were based on those suggested by two studies (17,18) and briefly as follows: i) no more than four mismatches between sRNA and the target (G-U bases count as 0.5 mismatches), ii) no more than two adjacent mismatches in the microRNA/target duplex, iii) no adjacent mismatches in positions 2–12 of the microRNA/target duplex (5′ of microRNA), iv) no mismatches in positions 10–11 of microRNA/target duplex, v) no more than 2.5 mismatches in positions 1–12 of the of the microRNA/target duplex (5′ of microRNA), vi) minimum free energy (MFE) of the microRNA/target duplex should be ≥75% of the MFE of the microRNA bound to its perfect complement. Subsequently, all target gene candidates were mapped to gene ontology (GO) terms, calculating gene numbers for each term, then using a hypergeometric test to identify significantly enriched GO terms in target gene candidates compared with the reference gene background (19).

## Results

### Sequencing and annotation of small RNAs

**Figure 1:**
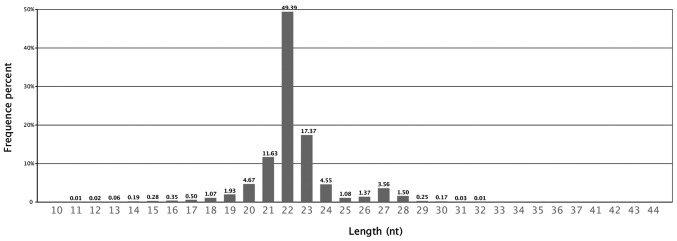
Distribution of small RNAs in the Klinefelter syndrome library.

**Figure 2:**
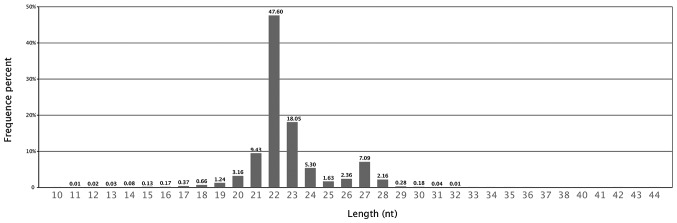
Distribution of small RNAs in the healthy control library.

In the two small RNA libraries, 9,993,746 and 9,864,510 high quality sequence reads were acquired by high-throughput sequencing from patients with Klinefelter syndrome and healthy controls, respectively. After filtering of the high-quality clean reads, 9,772,392 and 9,717,633 small RNA reads were obtained from patients with Klinefelter syndrome and healthy controls, respectively, and size distribution is shown in Figs. 1 and 2.

**Figure 3:**
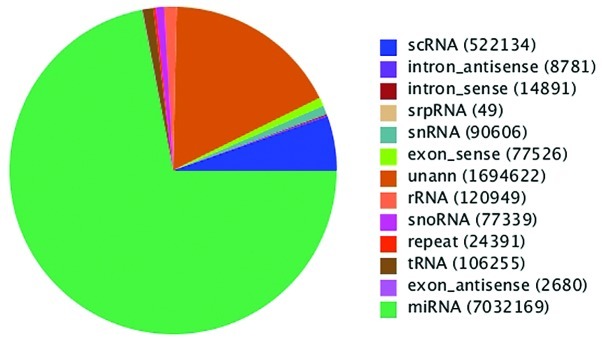
Annotation of small RNAs in the Klinefelter syndrome library.

**Figure 4:**
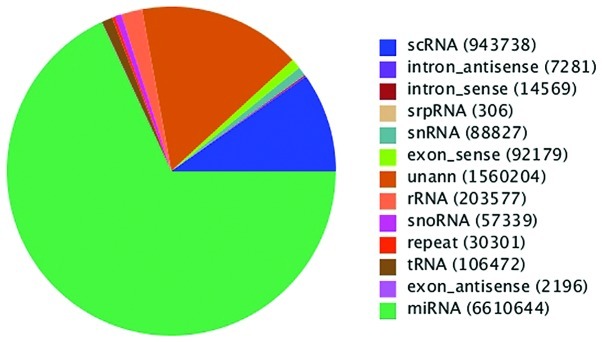
Annotation of small RNAs in the healthy control library.

There were 8,014,466 genome-matched reads (82.01%) for Klinefelter syndrome and 8,104,423 genome-matched reads (83.40%) for healthy controls, respectively, which were mapped to the human genome (hg19, National Center of Biotechnology Information build 37.1) using the SOAP program. They were divided into various categories of small RNAs according to their biogenesis and annotation shown in Figs. 3 and 4.

### Novel microRNAs prediction

Based on the sequences in the small RNA libraries, 64 and 49 novel microRNAs in the Klinefelter syndrome and healthy control libraries were identified, separately; 25 novel microRNAs were identified in both libraries (Table I). The most abundant novel microRNA sequence (CCCTGGGGTTCTGAGGACATG) of the Klinefelter syndrome library was also detected in the healthy control library.

### Differentially expressed patterns of known microRNAs

Compared with healthy controls, 71 microRNAs were expressed at a higher level in patients with Klinefelter syndrome and 395 microRNAs were expressed at approximately equal levels, while 18 microRNAs with reduced expression levels in Klinefelter syndrome were observed (Table II). Analysis of copy numbers of known microRNAs showed that there were various copy numbers of microRNAs between the two libraries. Among them, hsa-let-7f was the most prevalent microRNA in both the Klinefelter syndrome and healthy control libraries.

### Functional annotation of novel microRNA targets

There were 494,830 and 379,020 target genes of novel microRNAs in the Klinefelter syndrome library and the healthy control library. Clustering of over-represented GO classes in predicted targets of novel microRNAs showed that the most significant GO terms in the Klinefelter syndrome library (P<0.001) were genes involved in the endomembrane system (GO, 0012505), nucleotide binding (GO, 0000166), purine nucleotide binding (GO, 0017076), ribonucleotide binding (GO, 0032553), adenyl nucleotide binding (GO, 0030554), adenyl ribonucleotide binding (GO, 0032559), kinase activity (GO, 0016301) and binding (GO, 0005488).

## Discussion

Certain studies suggest that high-throughput sequencing is able to yield millions of clean reads per run (20). microRNAs are a large groups of small RNAs that play important roles in regulating gene expression and protein translation (21,22). To gain insight into the character of microRNAs in Klinefelter syndrome, we employed high-throughput sequencing technology to globally study small RNAs, particularly microRNA expression profiles in Klinefelter syndrome patients and their normal counterparts. We were able to obtain approximately 10 million 18–30 nt high quality sequence reads, which indicated that this platform clearly has great potential for the discovery of even rare microRNAs.

It is valuable to use high-throughput sequencing technology in the sequencing frequencies of microRNAs as an index for estimating the relative abundance of microRNAs, and the production of abundant microRNAs reads, allowing us to determine the differential expression of microRNAs (23). According to the sequencing frequencies, we first identified in-house differential expression of different microRNAs in Klinefelter syndrome, for example the sequence reads of hsa-let-7e is 1, and hsa-let-7f is 2, 291,067. The results suggested that various microRNAs showed obvious differential expression levels between patients with Klinefelter syndrome and healthy controls. Further sequence tag analysis revealed that 89 microRNAs were aberrantly expressed, 71 microRNA had increased expression and 18 microRNAs exhibited decreased expression, in Klinefelter syndrome compared with healthy controls. These results showed that upregulated microRNAs were more common than downregulation in Klinefelter syndrome. However, our data also revealed that certain microRNAs did not demonstrate differential expression between Klinefelter syndrome and healthy controls. We suggest that the expression of certain microRNAs is stable in Klinefelter syndrome and healthy controls, which may play an important function in the common physiological condition.

Thus far, knowledge concerning microRNAs in Klinefelter syndrome is limited. There are 77 microRNAs located on the X-chromosome, and 10 within the various functional parts of the brain, including let-7f-2, miR-19b-2, miR-92a-2, miR-98, miR-105-2, miR-221, miR222, miR363, miR374a, miR-374b, that may play roles in general intelligence (24). However, differential expression of these microRNAs could not be identified in PBMCs between Klinefelter syndrome and healthy control libraries in this study, which indicated that the expression pattern of microRNA varies over time and between tissues (25).

Klinefelter syndrome and healthy control libraries both contained multiple and heterogeneous small RNA species in the present study. The characteristic stem-loop hairpin secondary structure is an important feature for distinguishing microRNAs from other endogenous small RNAs (26). In total, 64 and 49 novel microRNAs were predicted in Klinefelter syndrome and healthy control libraries by providing evidence of stem-loop structures of the potential pre-microRNAs. Notably, the read numbers of the majority of novel microRNAs were much smaller compared with the known microRNAs, which indicated that novel microRNAs were usually expressed at lower levels. Certain novel microRNAs, for example microRNA sequences (AAACTGGGCATAGCTGTACTTTT, ACAGGGAGAGGAGGTAGAGGGA, ACGGTGGTGGTGG TGGTGGTGGTG and so on), could be identified in the Klinefelter syndrome library only, and it may be that certain marker microRNAs of Klinefelter syndrome require further study. In addition, our data revealed that there was one high abundance novel microRNA sequence (TTTGGGATTGACGCCACATGT) in the Klinefelter syndrome library, but not in the healthy control library. This further suggests that high-throughput sequencing is suitable for detecting novel microRNAs (14). GO analysis revealed that the most significant GO terms concerning microRNA targets in both libraries were genes involved in the endomembrane system, kinase activity and binding. Notably, studies have shown that certain binding proteins may be involved in the mechanisms of mammalian germ cell aneuploidy (27). Further research is required to elucidate whether these novel microRNAs are actually involved in that bioprocess.

The novel and known microRNAs in both libraries indicated that microRNAs could be mechanistically involved in the pathological process of Klinefelter syndrome. These microRNAs in PBMCs of Klinefelter syndrome require further validation individually in more specimens. Further studies of *in vitro* and *in vivo* models are necessary to clarify their roles in pathological processes. In summary, our data revealed the characterization of microRNA expression profiling in peripheral blood in Klinefelter syndrome, which may be valuable in the study of the effect of microRNAs on gene regulation in the future.

## Figures and Tables

**Table I:** Novel candidate microRNAs in Klinefelter syndrome and healthy control libraries.

Name	Sequence	Arm	Length	Location	Precursor length (nt)	MFE	Count
miRNA-01	AAAGGGGACAGCTCACAGGATT	5′	22	chr4:16229484:16229558:+	75	−22.2	9
miRNA-02	AAATGAATCATGTTGGGCCTGT	3′	22	chr10:115051380:115051452:-	73	−53.8	10
miRNA-03	AACAAGAGAGCAGAACGAGGTT	5′	22	chr6:6152172:6152250: −	79	−22.5	32
miRNA-04	AAGGTAGCGGGAGGGTTGGGCT	3′	22	chr3:45883723:45883820:+	98	−40.5	8
miRNA-05	ACTGGCAAAAGGGTTTAGAACT	5′	22	chr16:50830166:50830246:+	81	−20.34	30
miRNA-06	AGAGGAGGGTGGAGCAAGTGGT	5′	22	chr2:68622734:68622824:+	91	−31.8	40
miRNA-07	AGCAGAGACGTTGGAACTGGGCT	5′	23	chr1:150534435:150534511: −	77	−28.6	8
miRNA-08	CATGGCACTGGAGTAGAGCAT	3′	21	chr8:141181858:141181951: −	94	−33.5	14
miRNA-09	CCCTGGGGTTCTGAGGACATG	5′	21	chr9:35710651:35710743: −	93	−39	711
miRNA-10	GAGAGCTCCGACTGCAGCTGC	3′	21	chr11:133027217:133027311: −	95	−35.3	20
miRNA-11	GAGGACCCTGCAGGAATGGACG	5′	22	chr11:122650500:122650575:+	76	−33.7	10
miRNA-12	GATGAGGAGGATGAGGAGGATG	5′	22	chr6:3616133:3616211:+	79	−21.7	14
miRNA-13	GGAGGAACCTTGGAGCTTCGGCA	3′	23	chr22:31556037:31556127: −	91	−45.3	70
miRNA-14	TAAGCATGCTGTCTTTCTGGAG	3′	22	chr15:28518177:28518257: −	81	−20.8	7
miRNA-15	TAGGCCATTTTGGAAGCTGTTT	5′	22	chr2:33498389:33498471:+	83	−23	19
miRNA-16	TCAGGCCAGGCTGGGAGGATGC	5′	22	chr1:55784355:55784424:+	70	−52.4	8
miRNA-17	TCCTGGAGCTGGGCAGATGGGA	5′	22	chr19:2762628:2762702: −	75	−35.7	5
miRNA-18	TCCTGTACTGAGCTGCCCCGAGC	5′	23	chr8:41517952:41518035:+	84	−59.1	18
miRNA-19	TCGACTTGCTCGGGCCCGGCT	3′	21	chr10:104402512:104402584: −	73	−34	15
miRNA-20	TCGGGCGGGAGTGGTGGCTTTT	3′	22	chr6:28918819:28918903:+	85	−22.2	233
miRNA-21	TGGGCAGGGGCTTATTGTAGGAGT	5′	24	chr6:32137807:32137893: −	87	−30.1	97
miRNA-22	TGGGGCTGCGGTGGGTCGGGA	3′	21	chr21:45209209:45209298: −	90	−46.6	27
miRNA-23	TTGAGGGGAGAATGAGGTGGAGA	5′	23	chr1:43830309:43830391: −	83	−33.5	11
miRNA-24	TTGCTGGGAAAGGGAGAAGTTCAT	3′	24	chr8:1703286:1703370: −	85	−46.8	8
miRNA-25	TTGGAGGGTGTGGAAGACAT	5′	20	chr19:13051290:13051374:+	85	−31	10

Name, the name of novel microRNAs in Klinefelter syndrome and healthy control libraries; sequence, microRNA sequence cloned in the small RNA library; arm, the microRNA location in the predicted hairpin structure 5′ or 3′ arm; length, the length of microRNA (bp); location, microRNA location in the chromosome; precursor length, the length of precursor microRNA; MFE, minimum free energy; count, the counts of microRNA reads.

**Table II:** Differentially expressed known microRNAs between the Klinefelter syndrome and healthy control libraries.

Name	Klinefelter syndrome-std	Healthy control-std	Fold change (log_2_ ratio)	P-value
hsa-miR-451a	7844.0913	2482.9131	−1.65957256	0
hsa-miR-486	3190.7976	743.1139	−2.10226184	0
hsa-miR-27a	455.2549	982.3593	1.10957624	0
hsa-miR-144	320.4484	112.562	−1.5093722	1.57E-222
hsa-miR-181a-2	276.7135	609.6767	1.13965138	1.65E-273
hsa-miR-142	262.1009	537.0231	1.03486184	1.68E-206
hsa-miR-27b	215.5875	572.122	1.40804929	0
hsa-miR-4732	50.7325	6.3444	−2.99935462	4.17E-85
hsa-miR-181a	48.8802	129.1393	1.40160602	2.05E-81
hsa-miR-31	35.1938	107.6502	1.6129578	1.67E-83
hsa-miR-122	33.3415	71.528	1.10118909	5.80E-32
hsa-miR-1255a	32.5182	12.8935	−1.33460346	2.65E-20
hsa-miR-95	28.2991	59.3509	1.06851374	1.35E-25
hsa-miR-144	22.4334	45.9458	1.0342847	3.31E-19
hsa-miR-361	19.4492	46.8667	1.2688524	1.61E-26
hsa-miR-182	15.23	4.8095	−1.66295712	1.11E-13
hsa-miR-532	11.7313	35.6105	1.6019398	1.76E-28
hsa-miR-324	10.4964	23.638	1.17121337	1.26E-12
hsa-miR-34c	8.747	19.2379	1.13709112	4.06E-10
hsa-miR-369	7.4092	3.5815	−1.04875384	0.000291414
hsa-miR-1271	7.2034	15.1447	1.07206308	1.25E-07
hsa-miR-99a	6.7918	28.9591	2.09215089	4.44E-33
hsa-miR-365b	6.0714	1.8419	−1.72083449	1.75E-06
hsa-let-7i	5.8656	13.9168	1.24647692	1.10E-08
hsa-miR-329	5.8656	2.1489	−1.44868034	3.38E-05
hsa-miR-1306	4.7337	9.9259	1.06822964	2.04E-05
hsa-miR-193b	4.7337	12.2795	1.37521164	7.10E-09
hsa-miR-654	4.7337	2.2512	−1.07227404	0.003290144
hsa-miR-125b	4.6308	14.4284	1.63957797	8.65E-13
hsa-miR-449c	4.5279	0.8186	−2.46761152	2.13E-07
hsa-miR-873	4.322	9.5166	1.13874716	1.12E-05
hsa-miR-186	4.1162	12.3818	1.58883607	9.69E-11
hsa-miR-4433	3.6017	1.7396	−1.0499224	0.011952811
hsa-miR-454	3.6017	1.7396	−1.0499224	0.011952811
hsa-miR-548w	3.4988	8.1863	1.22635134	1.58E-05
hsa-miR-146b	3.3959	9.8236	1.53245784	2.04E-08
hsa-miR-151b	3.293	7.163	1.12116143	0.000171603
hsa-miR-548ak	3.293	7.9817	1.27729354	1.08E-05
hsa-miR-100	2.6755	6.7537	1.33586957	2.84E-05
hsa-miR-206	2.6755	64.979	4.60209311	2.00E-152
hsa-miR-874	2.5726	10.3352	2.00626724	3.88E-12
hsa-miR-548o	2.4697	5.0141	1.02165496	0.003642313
hsa-miR-140	2.3668	4.8095	1.02294912	0.004378249
hsa-miR-3934	2.161	5.4234	1.32749851	0.000193986
hsa-miR-196a	2.0581	6.4467	1.64724777	1.80E-06
hsa-miR-3614	1.9552	5.2188	1.41640192	0.000123491
hsa-miR-365a	1.7494	3.5815	1.03370374	0.013404561
hsa-miR-365b	1.7494	3.5815	1.03370374	0.013404561
hsa-miR-4425	1.7494	4.5025	1.36386608	0.000539221
hsa-miR-1256	1.6465	3.5815	1.12116143	0.008265541
hsa-miR-215	1.6465	4.8095	1.54648441	8.48E-05
hsa-miR-320d	1.6465	3.4792	1.0793531	0.011616255
hsa-miR-3158	1.5436	0.307	−2.32998839	0.004261655
hsa-miR-337	1.5436	0.4093	−1.91506838	0.011408876
hsa-miR-769	1.5436	3.3769	1.12940051	0.009947674
hsa-miR-4750	1.4407	0.5116	−1.49368178	0.040158862
hsa-miR-1273c	1.3378	3.5815	1.42070149	0.001500021
hsa-miR-3136	1.3378	3.4792	1.37889316	0.002226957
hsa-miR-3609	1.3378	0.2047	−2.70827944	0.004039068
hsa-miR-369	1.2349	2.5582	1.05073484	0.035018005
hsa-miR-5091	1.2349	2.8652	1.21424163	0.012106435
hsa-miR-876	1.2349	3.5815	1.53616972	0.000768989
hsa-miR-200b	1.132	2.3536	1.05599519	0.042625027
hsa-miR-1246	1.0291	0.2047	−2.32980017	0.021878979
hsa-miR-3613	1.0291	4.4002	2.09618592	3.73E-06
hsa-miR-378d	1.0291	6.0374	2.55254421	9.22E-10
hsa-miR-582	0.9262	2.7629	1.57678768	0.002709278
hsa-miR-873	0.9262	2.1489	1.21420269	0.030629647
hsa-miR-99b	0.9262	4.1955	2.17944709	3.72E-06
hsa-miR-141	0.8232	2.4559	1.57693693	0.004783616
hsa-miR-31	0.7203	2.1489	1.57692854	0.008496598
hsa-miR-3917	0.7203	2.6606	1.88508182	0.000870192
hsa-miR-548i	0.7203	2.8652	1.99196604	0.000332941
hsa-miR-671	0.7203	1.7396	1.2720858	0.044754756
hsa-miR-200a	0.6174	2.8652	2.21435846	0.000124782
hsa-miR-509-3	0.6174	2.3536	1.93059176	0.00150729
hsa-miR-3179	0.5145	1.4326	1.47739286	0.042651583
hsa-miR-1298	0.4116	1.3303	1.69243674	0.031813773
hsa-miR-188	0.4116	1.7396	2.07944073	0.004523166
hsa-miR-548h	0.4116	1.7396	2.07944073	0.004523166
hsa-miR-577	0.4116	1.3303	1.69243674	0.031813773
hsa-miR-375	0.3087	1.5349	2.31386728	0.004594046
hsa-miR-4736	0.3087	1.1256	1.86641685	0.036103136
hsa-miR-1468	0.2058	1.5349	2.89882978	0.001365699
hsa-miR-4716	0.2058	1.4326	2.79932096	0.002438799
hsa-miR-548ai	0.2058	1.1256	2.45137935	0.013318743
hsa-miR-570	0.2058	1.1256	2.45137935	0.013318743
hsa-miR-125b-2	0.1029	2.1489	4.38428346	6.07E-06
hsa-miR-330	0.01	1.1256	6.81455042	0.000505

Name, the name of known microRNAs in Klinefelter syndrome and healthy control libraries; Klinefelter syndrome-std, read counts of each identified microRNA in Klinefelter syndrome library normalized to the total number of small RNA reads, and multiplied by a constant of 1×10^6^; healthy control-std, read counts of each identified microRNA in Klinefelter syndrome library normalized to the total number of small RNA reads, and multiplied by a constant of 1×10^6^; fold-change (log_2_ ratio), fold-change (log_2_ Klinefelter syndrome/healthy control). P≤0.001 was considered to indicate a statistically significant result.
